# Botanical Report

**Published:** 1812-10

**Authors:** 


### BOTANICAL REPORT.

THE BOTANICAL MAGAZINE since our last Report contains: ALÖE *soccotrina,* var. *β.* The *purpurascens* of Haworth, and the new edition of Hortus Kewensis. To this plate is added a diminished outline of the whole plant, by which its habit is well explained. We have so often expressed our approbation of this plan, that we hope to see it more generally adopted. This is the plant that was thought to produce the succotrine alöes of the shops, but the best sort is supposed now to be the product of the alöe *spicata,* a species perhaps not yet seen in any of our collections.

HESPERANTHA *pilosa,* var. *α.* This is the hairy variety of the species, of which the smooth one has been before figured in the Botanical Magazine; the latter has smaller flowers, with a greenish tinge on the inner side, which the former wants. These plants are chiefly valuable for their agreeable scent during the night, but in this instance it is said to be not perceptible, except in a dry warm atmosphere.

TRICHONEMA *speciosum.* Ixia *Bulbocodium,* as it formerly stood, contained several distinct species. This is the second from the Cape of Good Hope that has been figured in the Magazine, besides an European one, of which it is probable there are still several that ought to be considered as distinct. The drawing of this plant was taken at Mr. Knight's Exotic Nursery, King's-road, Little Chelsea.

PODOLOBIUM *trilobum;* the Chorizema *trilobum* of Dr. Smith, and Pultenœa *ilicifolia* of the Botanist's Repository. The keel in the the flower of this plant is fully as long as the wings, but in Chorizema it is much shorter; there are moreover other characters drawn from the legumen which distinguish these two genera, so that, although we are somewhat jealous that Mr. Brown is rather too fond of subtle divisions, we cannot but go with him in the distinction here made. In referring to the Hortus Kewensis we observe that it is there called P. *trilobatum;* and at first supposed that Dr. Sims had committed a slight error in quoting it *trilobum,* but upon further examination we find that Dr. Smith has been erroneously quoted in the Hortus Kewensis, and his trivial name being evidently meant to be retained, *trilobum* was of course proper, not *trilobatum.*

PHILADELPHUS *inodorus.* A very fine shrub, native of North America, and well worthy of being more generally cultivated.

CEANOTHUS *americanus.* New Jersey tea, a shrub long known in our gardens, and esteemed for the delicacy of its flowers and foliage. In America it seems to have been valued for other qualities than merely pleasing the eye, having been used as a substitute for the Chinese herb, when that was a dearer and a scarcer article of luxury than it is at present in that country; its roots have also been applied to medicinal purposes, and the twigs to dye a cinnamon color.

AZALEA *indica;* a valuable acquisition, which has long been anxiously sought after by our collectors of curious and beautiful exotics. We have heard indeed, but we cannot vouch for the truth of the report, that the owner of the principal nursery in the country once offered a thousand pounds to any one who would bring him a living plant. There was formerly a very fine shrub of this Azalea in the garden of M. Jerome van Beverninck, in Holland, which flourished there for twelve years, and has been spoken of with rapturous admiration by the Dutch botanists of that day. Dr. Sims expresses his surprise that a plant so much admired, yet so easily propagated, and that had been cultivated so long in a well known garden, should be lost to the continent, and have been till now unseen in this country. The only way to account for this is by supposing that its owner, proud of being the possessor of such a rarity, cautiously guarded against its being communicated to others. The consequence was that his own tree perished, and with it the power of acquiring another, which could not have happened had a more liberal spirit induced the owner to have distributed it amongst his friends. Through this same foolish and illiberal desire of being sole possessors, how many valuable acquisitions are every year lost to this country! The drawing of this rare acquisition was made at Mr. Vere's, Kensington Gore, in whose collection it flowered in March last, for the first time probably in this country.

ALBUCO *setosa;* a plant of more singularity than beauty, easily distinguished from its congeners, by the divaricate peduncles and bristly margin to the scales of the bulb, yet Mr. Ker has a specific character of more than twenty lines; but this writer's characters are in fact descriptions, and without any attempt to seize such points only only as will serve to distinguish the particular species from the rest of the genus, which may sometimes be done by a few words better than it could by as many pages.

SPARAXIS *tricolor;* three varieties, one with a variegated flower, a most splendid plant, as gaudy as a parroquet.

GLADIOLUS *trichonemifolius;* a species nearly related to G. *tristis*, and like that, having very fragrant flowers.

LOBELIA *unidentata;* a beautiful trailing plant, flowering great part of the summer. By the name of *single-toothed* is meant that there is one tooth on each side of the leaf, which makes two to each leaf, hence Donn called it, in his catalogue, *bidentata.*

LASIOPETALUM *quercifolium;* a pretty little shrub from New South Wales. There is a particular dulness in this figure, arising from its being taken off in red, which has been done for the sake of representing the better the stellated pubescence of a reddish-brown color, but a little red added to the black would perhaps have succeeded better. As it is, the spirit of the figure is quite destroyed, and at the same time the pubescence is not well represented, being much too bright a red, and the color of the green is spoiled.

LASIOPETALUM *solanaceum;* another, but much more rare, species of the same genus as the last; neither can we praise the coloring of this, there is a general dulness prevailing, which we can hardly account for, except it arise from the extreme paleness of the ink, by which the impressions have been taken.

BARTONIA *decapetala.* This is a new genus, dedicated to Dr. B. S. Barton, professor of botany and natural history, in the university of Pennsylvania. This appears to be a very fine figure, though taken from dried specimens, the plant never having yet flowered in this country. An apology is made for thus deviating from the plan of the work, which professes to give drawings from living plants only. A desire to pay the compliment of naming so fine a plant in honor of the American professor, we suppose, has been the temptation to depart from the usual practice. Dr. Sims professes to owe the communication to Mr. Pursh, a German botanist, author of a new North-American Flora, now in the press. This plant was found by Mr. Thomas Nuttall, in the neighbourhood of the Missouri, on arid volcanic soil, and brought by him to this country.

